# Plasmid-mediated quinolone resistance determinants in quinolone-resistant *Escherichia coli* isolated from patients with bacteremia in a university hospital in Taiwan, 2001–2015

**DOI:** 10.1038/srep32281

**Published:** 2016-08-30

**Authors:** Cheng-Yen Kao, Hsiu-Mei Wu, Wei-Hung Lin, Chin-Chung Tseng, Jing-Jou Yan, Ming-Cheng Wang, Ching-Hao Teng, Jiunn-Jong Wu

**Affiliations:** 1 Department of Medical Laboratory Science and Biotechnology, College of Medicine, National Cheng Kung University, Tainan, Taiwan; 2 Institute of Clinical Medicine, College of Medicine, National Cheng Kung University, Tainan, Taiwan; 3 Division of Nephrology, Department of Internal Medicine, National Cheng Kung University Hospital, College of Medicine, National Cheng Kung University, Tainan, Taiwan; 4 Department of Pathology, National Cheng Kung University Hospital, National Cheng Kung University, Tainan, Taiwan; 5 Institute of Clinical Pharmacy and Pharmaceutical Sciences, College of Medicine, National Cheng Kung University, Tainan, Taiwan; 6 Institute of Molecular Medicine, College of Medicine, National Cheng Kung University, Tainan, Taiwan; 7 Department of Biotechnology and Laboratory Science in Medicine, School of Biomedical Science and Engineering, National Yang Ming University, Taipei, Taiwan

## Abstract

The aim of this study was to characterize fluoroquinolone (FQ)-resistant *Escherichia coli* isolates from bacteremia in Taiwan in 2001–2015. During the study period, 248 (21.2%) of 1171 isolates were identified as levofloxacin-resistant. The results of phylogenetic group analysis showed that 38.7% of the FQ-resistant isolates belonged to phylogenetic group B2, 23.4% to group B1, 22.6% to groupA, 14.9% to group D, and 0.4% belonged to group F. FQ-resistant isolates were highly susceptible to cefepime (91.5%), imipenem (96.0%), meropenem (98.8%), amikacin (98.0%), and fosfomycin (99.6%), as determined by the agar dilution method. β-lactamases, including *bla*_TEM_ (66.1%), *bla*_CMY-2_ (16.5%), *bla*_CTX-M_ (5.2%), *bla*_DHA-1_ (1.6%), and *bla*_SHV-12_ (1.6%), were found in FQ-resistant isolates. The results of PCR and direct sequencing showed that 37 isolates (14.9%) harbored plasmid-mediated quinolone resistance (PMQR) genes. *qnrB2*, *qnrB4*, *qnrS1*, coexistence of *qnrB4* and *qnrS1*, *oqxAB*, and *aac*(*6*′)-*Ib-cr* were found in 1, 4, 4, 1, 15, and 14 isolates, respectively. PMQR genes were successfully transfered for 11 (29.7%) of the 37 PMQR-harboring isolates by conjugation to *E. coli* C600. These findings indicate that *qnr* genes remained rare in *E. coli* but demonstrate the potential spread of *oqxAB* and *aac*(*6*′)-*Ib-c* in Taiwan.

Fluoroquinolones (FQs) are potent and broad-spectrum agents extensively used to treat a wide range of Gram-positive/negative bacterial infections by inhibiting the activity of both DNA gyrases (GyrA and GyrB) and the topoisomerase IV enzymes (ParC and ParE)^1^. Unfortunately, despite prescribing guidelines that now recommend reserving FQ use, over the last decade, worldwide spread of FQ-resistant organisms has reduced their therapeutic effectiveness and emerged as an important threat to global health^2^.

Organisms resistant to FQs can occur via several mechanisms, including intrinsic mutations under selection pressure or harboring transferable plasmid-mediated quinolone resistance (PMQR) determinants^2^. The most common mechanism of high-level FQ resistance is due to mutation in one or more of the genes that encode the targets of FQs. Kishii *et al.* showed the mutations that alter the expression and function of outer membrane protein, OmpF, can also lead to FQ resistance in *Escherichia coli*^3^. In addition, resistance can be conferred by upregulation of chromosomal multidrug efflux pumps (for example, AcrAB-TolC) (by mutations in regulatory proteins), increasing the capability of actively removing FQs and other drugs from the bacterial cell^4^.

Although FQ resistance can arise by a range of mechanisms, the greatest concern is placed on these bacteria harboring transferable PMQR genes; for example *qnr* alleles, *oqxAB*, *qepA*, and *aac(6′)Ib-cr*^5^,^6^,^7^,^8^. The binding of the Qnr protein to the topoisomerase physically prevents the intercalation of the FQs with the target enzyme and thus causes drug resistance^5^. A variant of an aminoglycoside acetyl transferase, *aac(6′)-lb-cr*, is able to confer decreased susceptibility to FQs by acetylating the amino nitrogen on the piperazinyl substituent present in these antimicrobial agents^6^. Moreover, two plasmid borne efflux systems, *oqxAB* and *qepA*, which encode transporters that can export FQs and other drugs, have become increasingly prevalent among *Enterobacteriaceae* over the past decade^7^,^8^.

Although most *E. coli* are harmless, some pathogenic *E. coli* isolates can cause diverse gastrointestinal or urinary tract diseases, and even bacteremia, and thus cause millions of death every year. The characterization of FQ-resistant *E. coli* was reported worldwide; however, isolates in most studies were enrolled over a relatively short duration. As a result, the longitudinal evolution and epidemiologic trends FQ-resistant *E. coli* isolates are possibly hidden. The aim of this study was to investigate the molecular epidemiology of FQ-resistant *E. coli* isolated from patients with bloodstream infections in Taiwan, 2001–2015.

## Results

### Long-term surveillance and antimicrobial susceptibility of FQ-resistant *E. coli*

During the study period, 2001–2015, we randomly selected 1,171 *E. coli* isolates from patients with bacteremia, of which 248 (21.2%) were identified as levofloxacin-resistant by using the disk diffusion method (Table 1). The trend in the prevalence of FQ-resistant invasive isolates remained stable during the 15-year surveillance (19.2–24.3%) (Table 1). The phylogenetic analysis revealed five groups (A, B1, B2, D, and F) in 248 FQ-resistant isolates. Ninty-six (38.7%) of the FQ-resistant isolates belonged to phylogenetic group B2. Phylogenetic group B1 was the second most common, representing in 23.4% of the isolates, followed by group A (22.6%), group D (14.9%), and group F (0.4%) (Table 1). The dramatically increasing ratio of phylogenetic group B2 among FQ-resistant isolates was revealed during the study period (Table 1).

**Figure 1:**
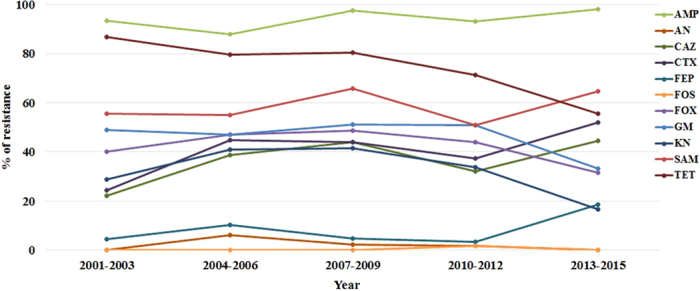
Trends of antimicrobial resistance among 248 FQ-resistant *E. coli*, 2001–2015. AMP, ampicillin; AN, amikacin; CAZ, *ceftazidime*; CTX, *cefotaxime*; FEP, cefepime; FOS, fosfomycin; FOX, cefoxitin; GM, gentamicin; KN, kanamycin; SAM, ampicillin-sulbactam; TET, tetracycline.

The susceptibilities of the 248 FQ-resistant isolates to 15 antimicrobial agents are shown in Table 2. All isolates were resistant to levofloxacin and ciprofloxacin, as determined by the agar dilution method. However, the entire collection was highly susceptible to cefepime (91.5%), imipenem (96.0%), meropenem (98.8%), amikacin (98.0%), and fosfomycin (99.6%) (Table 2). One isolate showed resistance to tigecycline, and all isolates were susceptible to colistin. Moreover, a total of 89 (35.9%) and 223 (89.9%) isolates were defined to be ESBL-producers and multidrug resistant (MDR) strains, respectively. The trends of resistance of FQ-resistant invasive isolates to 11 selected antimicrobial agents were generally stable during this 15-year surveillance (Fig. 1). The prevalence of antimicrobial resistance to tetracycline decreased from 86.7% to 55.6% during this period (Fig. 1).

### Characterization of antimicrobial resistance genes

The numbers of β-lactamase- and PMQR-producers among the 248 FQ-resistant isolates are shown in Table 1. The results showed that the dominant β-lactamase was *bla*_TEM_ (66.5%), followed by *bla*_CMY_ (19.0%), *bla*_CTX-M_ (4.8%), *bla*_DHA_ (1.6%), and *bla*_SHV_ (1.6%) in FQ-resistant *E. coli* isolates (Table 1). Sequence analysis revealed that 6 *bla*_CTX-M-14_, 3 *bla*_CTX-M-174_, 2 *bla*_CTX-M-15_, 1 *bla*_CTX-M-13_, and 1 *bla*_CTX-M-55_ genes were identified among 12 isolates producing *bla*_CTX-M_ type extended spectrum β-lactamases (ESBLs) (isolate 1902 harbored *bla*_CTX-M-14_ and *bla*_CTX-M-15_). Only the *bla*_SHV-12_ ESBL was found in 4 *bla*_SHV_-porducers. In addition, all *bla*_CMY_ and *bla*_DHA_ genes were identified as *bla*_CMY-2_ and *bla*_DHA-1_, respectively.

The prevalence of PMQR genes, including *qnr* alleles, *aac*(*6*′)-*Ib-cr*, *qepA*, and *oqxAB* were determined by PCR and direct sequencing, and the results showed that 37 FQ-resistant isolates (14.9%) harbored at least one PMQR gene (Table 1). *qnrB2*, *qnrB4*, *qnrS1*, and the coexistence of *qnrB4* and *qnrS1* were found in 1, 4, 4, and 1 isolates, respectively (Table 1). *oqxAB* and *aac*(*6*′)-*Ib-cr* genes were identified in 15 (isolate 1315 harbored only *oqxA* but not *oqxB*) and 14 isolates (2 isolates also harbored *qnrB4*), respectively (Table 1). *qnr* alleles, including *qnrA*, *qnrC*, *qnrD*, *qnrVC*, and *qepA*, were not found in any of the detected isolates. This survey also showed a trend of increase in the prevalence of *aac*(*6*′)-*Ib-cr* and *oqxAB* among FQ-resistant isolates between 2004–2006 and 2010–2012 (Table 1). Among 10 *qnr*-producers, *bla*_SHV-12_, *bla*_DHA-1_ *bla*_CMY-2_ were found in 3, 4, and 2 isolates, respectively. However, no *bla*_SHV-12_ or *bla*_DHA-1_ were detected in *oqxAB*- or *aac*(*6*′)-*Ib-cr-*producers. In contrast, *bla*_CMY-2_ was found in 7 *oqxAB*-producers (7/15, 46.7%) and 6 *aac*(*6*′)-*Ib-cr*-producers (6/14, 42.9%), respectively.

### Characterization of QRDR mutations in PMQR-harboring isolates

Thirty-seven PMQR-harboring *E. coli* isolates were distributed into each of the four main phylogroups: A, 12 isolates (32.4%); B1, 15 isolates (40.5%); B2, 6 isolates (16.2%); and D, 4 isolates (10.9%) (Table 3). Chromosomal QRDR mutations were determined by PCR and direct sequencing, and the results showed that only 1 (isolate 1019) and 2 (isolate 1706 and 1763) isolates contained wild-type GyrA and ParC, respectively (Table 3). The most common point mutations in PMQR-harboring isolates were GyrA S83L/D87N (31 isolates, 83.8%) and S83L (4 isolates, 10.8%), and those in ParC were S80I (23 isolates, 62.2%) and S80I/E84V (6 isolates, 16.2%) (Table 3).

### PMQR gene transfer and plasmid analysis

*E. coli* isolates harboring PMQR genes were further analyzed by conjugation tests to determine whether there was horizontal plasmid spread in Taiwan. Transfer of PMQR genes by conjugation to recipient cells of *E. coli* C600 was successful for 11 (29.7%) of the 37 selected isolates (2, 4, 1, 2, and 2 parental isolates harbored *qnrB*, *qnrS*, *qnrB*/*qnrS*, *oqxAB*, and *aac*(*6*′)-*Ib-cr*/*qnrB*, respectively) (Table 4). Plasmid numbers and sizes present in parental isolates and transconjugants were verified according to the method of Kado and Liu^9^, and the results showed that 14 transconjugants (except 1962-3) contained only a single plasmid with a size over 50 kb (Table 4). The antimicrobial resistance genes in transconjugants were further verified by PCR, and the results showed that the *aac*(*6*′)-*Ib-cr* and *qnrB4* genes were located on the same plasmid in 1377-3. Two and three transconjugants harboring different plasmid profiles were selected from parental isolates 1426 and 1962, respectively (Table 4). No co-transference of *qnrB4* and *qnrS1* were found in 30 randomly selected transconjugants from isolate 1426. In contrast, transference of *aac*(*6*′)-*Ib-cr*, *qnrB*, and *aac*(*6*′)-*Ib-cr*/*qnrB* from isolate 1962 was found in 6 (20%), 4 (13.3%), and 20 (66.7%) of transconjugants.

Co-transference of *bla*_DHA-1_ and *qnrB* to recipient cells was found in 3 of 5 *qnrB*-producers (isolates 1377, 1426, and 1962) (Table 4). No *bla*_CMY-2_ was detected in *oqxAB*- or *aac*(*6*′)-*Ib-cr-*harboring transconjugants. Transconjugant 1649-2 showed resistance to ampicillin and cefoxitin with an un-identified β-lactamase gene. In addition, transconjugants 1377-3, 1706-2, and 1962-2 showed increased MICs to tetracycline. The results indicated the co-transference of the tetracycline resistance gene with PMQR determinants. Moreover, 6 of 11 transconjugants showed high resistance to trimethroprim (MIC > 256 μg/mL) (Table 4). PCR-based replicon typing results revealed that IncN, IncFII, and IncHII were identified in 4, 4, and 2 PMQR-plasmids of transconjugants harboring only a single plasmid. However, 3 plasmids (613-3, 1377-3 and 1426-4) were nontypable by PCR-based replicon typing (Table 4).

## Discussion

In this study, we present the characteristics of 248 FQ-resistant bacteremia isolates of *E. coli* from Taiwan, 2001–2015. Among them, 37 isolates harbored at least one PMQR gene. *oqxAB* and *aac*(*6*′)-*Ib-cr* genes were most prevalent among PMQR-producers. In addition, horizontal transmission of PMQR genes is often accompanied by transmission of genes conferring resistance to other antimicrobial agents.

Antimicrobial resistance in Gram-negative bacteria is on the rise worldwide, particularly in *E. coli*, which constitutes a majority of invasive Gram-negative isolates. Wong *et al.* showed that ciprofloxacin resistance in *E. coli* isolated from bacteremia in Canada peaked in 2006 at 40% and subsequently stabilized at 29% in 2011, corresponding to decreasing ciprofloxacin usage after 2007^10^. In this study, we showed the prevalence of FQ-resistant invasive *E. coli* isolates is lower compared with Canada (Table 1). In addition, the prevalence of FQ resistance in bacteremia-causing *E. coli* was lower than urinary-tract-related *E. coli* in Taiwan (21.2% vs. 32%)^11^. Moreover, the entire collection was highly susceptible to cefepime, imipenem, meropenem, amikacin, and fosfomycin (Table 2). Fosfomycin is found active against *Enterobacteriaceae*, particularly *E. coli*, regardless of source (urinary tract infections or bacteremia), ciprofloxacin resistance, and ESBL production^12^,^13^,^14^. In addition, fosfomycin is recommended as one of the first-line agents for treatment of urinary tract infections (UTIs) in the latest guidelines endorsed by the Infectious Diseases Society of America and the European Society for Clinical Microbiology and Infectious Diseases^15^. As a result, the clinical usefulness of fosfomycin, as a first-line treatment agents of bacteremia *E. coli* infections, should be evaluated further, especially in regions where ciprofloxacin resistance rates are high.

The phylogenetic group B2 was the most common pathogenic *E. coli* in many countries, and group A and group B1 were usually isolated as commensals^16^,^17^. Massot *et al.* showed a parallel and linked increase in the frequency of the B2 group strains (from 9.4% in 1980 to 22.7% in 2000 and 34.0% in 2010) and of virulence factors^18^. Here, we showed 38.7% of the FQ-resistant bacteremia *E. coli* isolates belonged to phylogenetic group B2, followed by group B1 (23.4%), group A (22.6%), group D (14.9%), and group F (0.4%) (Table 1). Moreover, based on the 15-year epidemiologic analysis, we further showed that the increasing trend of group B2 among bacteremia *E. coli* isolates (Table 1). Phylogenetic group B2 dominates the bacteremia *E. coli* isolates during the period 2007–2015, but group B1 was most prevalent among bacteremia *E. coli* isolates during the period 2001–2006 (Table 1). As a result, the longitudinal collection of clinical isolates provides the opportunity to characterize the dynamics of the epidemiologic trend and evolution in infectious pathogens over long periods.

Zhao *et al.* showed that *qnr*, *aac*(*6*′)*-Ib-cr*, *qepA*, and *oqxAB* were found in 2.7%, 24.5%, 11.9% and 6.3% of ciprofloxacin-resistant *E. coli* isolates in China, respectively^19^. Yang *et al.* showed that PMQR genes were detected in 59 of 80 (73.8%) ciprofloxacin-nonsusceptible bacteremia *E coli* isolates from Korea^20^. In this study, we revealed the prevalence of PMQR genes among FQ-resistant *E. coli* in Taiwan (14.9%) was relatively lower than in China (37.3%)^19^ or in Korea (73.8%)^20^. In addition, the dominant PMQR genes among FQ-resistant *E. coli* in Taiwan is *oqxAB* (40.5%), followed by *aac*(*6*′)-*Ib-cr* (37.8%), and *qnr* alleles (27.0%). No *qepA*-producer was found in this study. Although PMQR genes provide a low level of FQ resistance, they have been reported to favor the selection of additional chromosome-encoded resistance mechanisms^21^. Moreover, all of the PMQR-positive isolates had QRDR mutations (Table 3). These results suggest that along with high-level resistance mediated by QRDR mutations, selection pressure from FQs was absent, and in this case PMQR genes may be lost^21^. It is possible that evolution by natural selection may explain the higher level of FQ resistance and the relatively lower prevalence of PMQR genes in FQ-resistant invasive *E. coli* from Taiwan. As a result, continual epidemiologic surveillance of PMQR genes is necessary to evaluate whether there are specific plasmids disseminated in Taiwan.

Previous studies showed the most common point mutations in ciprofloxacin-resistant *E. coli* isolates from China were GyrA S83L/D87N (263 isolates, 87.1%) and S83L (21 isolates, 7.0%), and those in ParC were S80I (233 isolates, 77.2%) and S80I-E84V (35 isolates, 11.6%)^19^. Our results regarding the distribution of QRDR mutations among FQ-resistant isolates were consisted with previous studies (Table 3). Isolate 1019 showed low-level FQ resistance presented S129A/S134G/A141V/L151M substitutions in ParC in the absence of GyrA substitutions raised the possibility that these mutations were not associated with FQ resistance. However, the direct evidence to demonstrate the association of specific QRDR mutations with FQs susceptibility is still limited and thus worth investigating.

A striking association between *bla*_DHA-1_ and *qnrB4* was reported in Korea and Taiwan^22^,^23^, and this tight association was also observed in our study (Table 4). The co-transference of the *bla*_DHA-1_ and *qnrB4* genes was identified by conjugation assay (3/4, 75%) (Table 4). In contrast, although 7 *oqxAB*- (7/15) and 6 *aac*(*6*′)-*Ib-cr*-producers (6/14) also carried *bla*_CMY-2_, the results of the conjugation assay showed that no *bla*_CMY-2_ was located on *oqxAB*- or *aac*(*6*′)-*Ib-cr*-containing plasmids (Table 4). To our knowledge, this is the first description of the high co-occurrence of *bla*_CMY-2_ in *oqxAB* or *aac*(*6*′)-*Ib-cr*-producing *E. coli*.

Highly transferable PMQR genes were observed in this study (11/37, 29.7%) (Table 4). Additional phenotypically expressed resistances were co-transferred with PMQR genes by 12 plasmids (92.3%, except 613-3), resulting in diverse resistance patterns (Table 4). Overall, the most frequently co-transferred resistances were to ampicillin (69.2%), trimethoprim (42.6%), ceftazidime (38.5%), cefotaxime (30.8%), cefoxitin (30.8%), kanamycin (30.8%), and tetracycline (23.1%) (MICs > 4-fold change) (Table 4). These results indicated the high co-existence of antimicrobial resistance genes on the PMQR-plasmids.

In summary, plasmid profiling of *E. coli* isolates exhibiting the co-existence of both PMQR genes and other antimicrobial resistance genes on a single plasmid shows how they contribute to the rapid spread and increase in bacterial resistance, which is important to public health. The plasmid backgrounds of the PMQR genes were variable, ruling out the hypothesis for the spread of specific plasmids in Taiwan, however, continual epidemiologic surveillance and monitoring antimicrobial prescriptions and consumption would decrease the prevalence of FQ-resistant organisms and PMQR spread.

## Methods

### Sampling and isolation of ***E. coli***

Bacteremia *E. coli* isolates were recovered in National Cheng Kung University hospital, 2001 to 2015. The Ethics Committee approved that no formal ethical approval was needed to use these clinically obtained materials, because the isolates were remnants from patient samples, and the data were analyzed anonymously. A total of 1,171 non-duplicate clinical isolates were randomly selected and stored at −80 °C in Luria-Bertani (LB) broth containing 20% glycerol (v/v) until used. *E. coli* was identified in the clinical laboratory by colony morphology, Gram stain, biochemical tests, and the Vitek system (bioMérieux, Marcy l′Etoile, France) according to the manufacturer’s recommendations. Susceptibility to levofloxacin for *E. coli* isolates was determined by the disk diffusion method (5 μg/disc, *BD BBL*™ Sensi-*Disc*™, Sparks, MD, USA) on Mueller-Hinton (MH) agar (Bio-Rad, Marne la Coquette, France) based on the CLSI guidelines^24^. A total of 248 levofloxacin-nonsusceptible bacteremia *E. coli* isolates were identified for further analysis.

### Antimicrobial susceptibility testing

Antimicrobial susceptibilities to ampicillin, ampicillin-sulbactam, gentamicin, colistin, and tigecycline (BD BBL™ Sensi-Disc™) were determined by the disk diffusion method on Mueller-Hinton agar^24^. MICs of selected antimicrobial agents (from Sigma-Aldrich: amikacin, cefepime, cefotaxime, ceftazidime, ciprofloxacin, fosfomycin, kanamycin, levofloxacin; from USP Standards: cefoxitin, imipenem, meropenem) were determined by the agar dilution method in accordance with CLSI guidelines^24^. *E. coli* ATCC 25922 and *Pseudomonas aeruginosa* ATCC 27853 were used as quality control strains. The interpretation of resistance to these antimicrobial agents was determined according to the recommendations of the CLSI^25^. Tigecycline and colistin susceptibilities were interpretated according to the European Committee on Antimicrobial Susceptibility Testing (EUCAST)^26^ and previous study^27^, respectively. MDR *E. coli* was defined as isolates that were resistant to at least 3 classes of the tested antimicrobial agents^28^.

### Characterization of antimicrobial resistance genes

All 248 FQ-resistant *E. coli* isolates were further screened for selected β-lactamases (*bla*_TEM_, *bla*_SHV_, *bla*_CTX-M_, *bla*_DHA_, and *bla*_CMY_) and PMQR genes (*qnr* alleles, *oxqAB*, *qepA*, and *aac(6′)Ib-cr*) by PCR amplification with specific primers (Supplmentary Table S1). DNA sequencing was further carried out on β-lactamases (except *bla*_TEM_) and PMQR genes, and the DNA sequences and deduced amino acid sequences were compared with genes in the GenBank database (http://www.ncbi.nlm.nih.gov/genbank/) to confirm the subtypes of antimicrobial resistance genes.

### Screening for mutations in quinolone resistance-determining regions

GyrA and ParC QRDRs of 37 isolates harboring PMQR genes were examined by amplifying and sequencing *gyrA* (490 bp) and *parC* (470 bp) genes using primers (Supplmentary Table S1) described by Zhao *et al.*^19^. Amplimers were sequenced and amino acid mutations were determined using the control strain *E. coli* K-12 (NZ_AKBV01000001.1) as a reference.

### Determination of the phylogenetic origin of *E. coli* isolates

Phylogenetic grouping of *E. coli* isolates was performed using a previously published method^29^. Primers used are described in Supplmentary Table S1. The PCR-amplified products were separated by electrophoresis on 1.8% agarose gels, stained with ethidium bromide, and assigned to one of the seven phylogenetic groups A, B1, B2, C, D, E and F.

### Conjugation experiments and plasmid analysis

The liquid mating-out assay was carried out to transfer PMQR genes from 37 FQ-resistant *E. coli* isolates to rifampicin-resistant *E. coli* C600 as described previously^30^. Transconjugants were selected on LB plates containing 256 μg/mL rifampicin (Sigma) and 0.06 μg/mL ciprofloxacin. The plasmids were extracted as described previously^9^, followed by electrophoresis in a 0.6% agarose gel at 50 V for 3 h and compared by co-electrophoresis with plasmids of known sizes from *Salmonella* OU7526 and a GeneRular^TM^ DNA ladder (Fermentas, Burlington, ON, Canada) to predict the plasmid sizes^30^. Plasmids were typed by PCR-based replicon typing according to the previous study^31^.

## Additional Information

**How to cite this article**: Kao, C.-Y. *et al.* Plasmid-mediated quinolone resistance determinants in quinolone-resistant *Escherichia coli* isolated from patients with bacteremia in a university hospital in Taiwan, 2001–2015. *Sci. Rep.* **6**, 32281; doi: 10.1038/srep32281 (2016).

## Supplementary Material

Supplementary Information

## Figures and Tables

**Table 1:** Distribution of phylogenetic group, PMQR genes, and β-lactamase genes in 248 FQ-resistant bacteremia *E. coli* isolates.

Characteristic	No. (%) of isolates					
	2001–2003 45 (19.4)	2004–2006 49 (20.4)	2007–2009 41 (19.2)	2009–2012 59 (24.3)	2013–2015 54 (22.2)	Total 248 (21.2)
Phylogenetic group, No. (%)						
A	10 (22.2)	14 (28.6)	11 (26.8)	14 (23.7)	7 (13.0)	56 (22.6)
B1	16 (35.6)	14 (28.6)	10 (24.4)	8 (13.6)	10 (18.5)	58 (23.4)
B2	7 (15.5)	7 (14.2)	18 (44.0)	29 (49.1)	35 (64.8)	96 (38.7)
D	12 (26.7)	14 (28.6)	1 (2.4)	8 (13.6)	2 (3.7)	37 (14.9)
F	0	0	1 (2.4)	0	0	1 (0.4)
PMQR genes, No. (%)^a^						
*qnrB*	—	1 (*qnrB2*) 1 (*qnrB4*)	—	1 (*qnrB4*)	—	3
*qnrS*	1 (*qnrS1*)	—	—	1 (*qnrS1*)	2 (*qnrS1*)	4
*qnrB* + *qnrS*	—	—	—	1 (*qnrB4*, *qnrS1*)		1
*oqxAB*	2	6	2^b^	4	1	15
*aac*(*6*′)-*Ib-cr*	—	4	2	5	1	12
*qnrB* + *aac*(*6*′)-*Ib-cr*	—	—	1 (*qnrB4*)	—	1 (*qnrB4*)	2
β-lactamase genes, No. (%)						
*bla*_TEM_	28	21	21	27	28	125
*bla*_CTX-M_	0	0	0	0	7	7
*bla*_CMY_	0	4	1	8	1	14
*bla*_TEM_/*bla*_CTX-M_	1	1	0	0	1	3
*bla*_TEM_/*bla*_DHA_	0	0	1	0	0	1
*bla*_TEM_/*bla*_CMY_	1	7	9	6	9	32
*bla*_TEM_/*bla*_SHV_	0	1	0	1	0	2
*Bla*_CTX_/*bla*_CMY_	0	0	0	0	1	1
*bla*_SHV_/*bla*_DHA_	0	0	0	1	0	1
*bla*_TEM_/*bla*_SHV_/*bla*_DHA_	0	1	0	0	0	1
*bla*_TEM_/*bla*_CTX-M_/*bla*_DHA_	0	0	0	0	1	1

^a^*qnr* alleles (*qnrA*, *qnrC*, *qnrD*, *qnrVC*) and *qep*A were not found in any of the detected isolates.

^b^Isolate 1315 harbored only *oqxA* but not *oqxB.*

**Table 2:** *In vitro* activity of 15 antimicrobial agents against 248 FQ-resistant bacteremia *E. coli* isolates.

Antibiotic^a^	MIC (μg/mL)			% Susceptibility		
	Range	MIC_50_	MIC_90_	S	I	R
Ampicillin^b^	—	—	—	6.0	1.2	92.8
Ampicillin-sulbactam^b^	—	—	—	42.0	17.0	41.0
Ceftazidime	0.06–>256	1	256	63.7	1.6	34.7
Cefepime	<0.03–>256	0.12	4	91.5	3.2	5.3
Cefotaxime	<0.03–>256	0.25	64	59.3	1.6	39.1
Cefoxitin	0.12–>256	8	256	54.0	1.6	44.4
Imipenem	0.12–>256	0.25	0.5	96.0	2.8	1.2
Meropenem	<0.03–>128	<0.03	0.06	98.8	0	1.2
Amikacin	1–>256	4	8	98.0	0.4	1.6
Gentamicin^b^	—	—	—	54.0	3.6	42.4
Kanamycin	2–>256	16	>256	68.1	5.6	26.3
Tetracycline	1–>256	128	256	26.2	0.4	73.4
Fosfomycin	0.25–>256	1	2	99.6	0	0.4
Ciprofloxacin	0.12–>256	32	128	0	1.6	98.4
Levofloxacin	4–128	16	64	0	3.6	96.4

MIC_50/90_, minimum inhibitory concentration for 50% and 90% of the isolates, respectively; S, susceptible; I, intermediate resistant; R, resistant.

^a^One isolate was resistant to tigecycline, and all isolates were susceptible to colistin.

^b^Antimicrobial susceptibilities of ampicillin, ampicillin-sulbactam, and gentamicin were determined by the disk diffusion method.

**Table 3:** Phylogenic group, MICs, PMQR genes and QRDR mutations of 37 *E. coli* isolates harboring PMQR genes.

Isolate	Year	Phylogenic group	MIC (μg/mL)		PMQR genes	QRDR mutations^a^	
			CIP	LVX		GyrA	ParC
534	2001	B1	64	32	*oqxAB*	S83L, D87N	S80I
613	2002	B1	4	8	*qnrS1*	S83L	A81P
680	2002	A	128	64	*oqxAB*	S83L, D87N	S80I, A108V
905	2005	B1	64	64	*oqxAB*	S83L, D87N	S80I
906	2005	B1	64	32	*oqxAB*	S83L, D87N	S80I
946	2005	A	256	64	*oqxAB*	S83L, D87N	S80I
966	2005	B1	128	32	*oqxAB*	S83L, D87N	S80I
970	2005	B1	64	32	*oqxAB*	S83L, D87N	S80I
977	2005	A	16	8	*oqxAB*	S83L, D87N	S80I
1019	2006	A	1	4	*qnrB2*	—b	S129A, S134G, A141V, L151M
1029	2006	A	128	64	*aac*(*6*′)*-Ib-cr*	S83L, D87N	S80I, E84G
1045	2006	D	128	16	*aac*(*6*′)*-Ib-cr*	S83L, D87N	S80I
1050	2006	A	16	32	*qnrB4*	S83Y	G78C, S129A, S134G A141V, L151M
1077	2006	D	128	8	*aac*(*6*′)*-Ib-cr*	S83L, D87N	S80I
1078	2006	D	256	16	*aac*(*6*′)*-Ib-cr*	S83L, D87N	S80I
1206	2007	B1	128	64	*oqxAB*	S83L, D87N	S80I
1262	2008	B2	128	16	*aac*(*6*′)*-Ib-cr*	S83L, D87N	S80I, E84V
1270	2008	B2	128	16	*aac*(*6*′)*-Ib-cr*	S83L, D87N	S80I, E84V
1315	2009	B1	8	8	*oqxA*	S83L, D87N	S80I
1377	2009	B1	128	64	*qnrB4*, *aac*(*6*′)*-Ib-cr*	S83L, D87N	S80I
1426	2010	B2	256	128	*qnrB4*, *qnrS1*	S83L, D87N	S80I, E84V
1465	2010	A	128	32	*oqxAB*	S83L, D87N	S80I
1480	2010	B1	64	32	*oqxAB*	S83L, D87N	S80I
1504	2011	A	128	32	*aac*(*6*′)*-Ib-cr*	S83L, D87N	S80I
1510	2011	A	128	32	*aac*(*6*′)*-Ib-cr*	S83L, D87N	S80I
1516	2011	B1	16	16	*qnrS1*	S83L	S80I
1540	2011	B2	256	16	*aac*(*6*′)*-Ib-cr*	S83L, D87N	S80I, E84V
1558	2011	B1	32	16	*oqxAB*	S83L, D87N	S80I
1604	2012	B1	128	64	*oqxAB*	S83L, D87N	S80I
1619	2012	B2	64	16	*aac*(*6*′)*-Ib-cr*	S83L, D87N	S80I, E84V
1649	2012	D	128	64	*qnrB4*	S83L, D87N	S80I
1705	2012	A	>256	64	*aac*(*6*′)*-Ib-cr*	S83L, D87N	A108T
1706	2013	B1	16	8	*qnrS1*	S83L	—b
1763	2013	A	32	64	*qnrS1*	S83L	—b
1878	2014	B1	16	16	*oqxAB*	S83L, D87N	S80I
1902	2015	B2	256	32	*aac*(*6*′)*-Ib-cr*	S83L, D87N	S80I, E84V
1962	2015	A	128	32	*qnrB4*, *aac*(*6*′)*-Ib-cr*	S83L, D87N	S80I

Abbreviations: CIP, ciprofloxacin; LVX, levofloxacin.

^a^QRDRs of *E. coli* K-12 (NZ_AKBV01000001.1) as a wild-type reference.

^b^Isolates with no mutations in the GyrA or ParC.

**Table 4:** MICs, antimicrobial resistance genes and plasmid profiles of *E. coli* isolates used in conjugation experiments.

Group and isolate	MIC (μg/mL) of antimicrobial agent										Presence or absence of PMQR genes				Plasmid characterization		
	AMP	CAZ	CTX	FOX	TET	KN	FOS	CIP	LVX	TMP	*qnrB*	*qnrS*	*aac*(*6′*)- *Ib-cr*	*oqxAB*	β-lactamase	No, Size (~Kb)^a^	Replicon(s)
Clinical isolates (donors)																	
534	>256	0.5	0.06	4	2	4	0.5	32	32	1	**−**	**−**	**−**	**+**	*bla*_TEM_	2, 50/80	IncFIB, IncFII, IncN
613	8	0.25	0.06	4	128	8	1	4	8	0.25	**−**	+(S1)	**−**	**−**	**−**	3, 60/80/>90	IncFIB, IncFII
906	>256	256	32	256	128	8	1	8	8	>256	**−**	**−**	**−**	**+**	*bla*_TEM_, *bla*_CMY-2_	4, 7/70/80/90	IncFIB, IncI1, IncFII
1019	>256	256	16	256	8	>256	32	1	2	>256	+(B2)	**−**	**−**	**−**	*bla*_TEM_, *bla*_SHV-12_	1, >90	IncHI2
1377	>256	64	8	256	128	>256	1	16	8	>256	+(B4)	**−**	**+**	**−**	*bla*_TEM_, *bla*_DHA-1_	10, 5/6/7/8/10/50/55/80/90/>90	IncFIB, IncI1, IncFIA
1426	>256	>256	32	>256	4	>256	1	256	256	>256	+(B4)	+(S1)	**−**	**−**	*bla*_SHV-12_, *bla*_DHA-1_	6, 7/15/23/50/90/>90	IncHI2, IncN
1516	>256	64	16	64	2	8	0.5	16	16	>256	**−**	+(S1)	**−**	**−**	*bla*_CMY-2_	7, 5/7/8/9/23/50/90	IncI1, IncFIC, IncFII, IncN
1649	>256	64	8	256	2	>256	1	64	64	>256	+(B4)	**−**	**−**	**-**	**−**	4, 7/23/80/>90	IncFII
1706	>256	0.5	0.06	2	64	8	4	2	8	0.25	**−**	+(S1)	**−**	**−**	*bla*_TEM_	5, 5/6/8/80/>90	IncFII,
1763	>256	128	8	128	>256	>256	1	32	64	>256	**−**	+(S1)	**−**	**−**	*bla*_TEM_, *bla*_CMY-2_	6, 8/10/55/70/90/>90	IncI1, IncFII,
1962	>256	64	>256	256	128	16	1	128	32	>256	+(B4)	**−**	**+**	**−**	*bla*_TEM_, *bla*_DHA-1_ *bla*_CTX-M-14_	8, 5/6/8/9/50/80/90/>90	IncHI2, IncFIB, IncFII, IncFIA, IncN
Recipient																	
C600	4	0.25	0.06	2	2	8	2	<0.03	0.06	0.25	**−**	**−**	**−**	**−**		**−**	**−**
Transconjugants																	
534-3	>256	0.25	0.06	2	2	8	2	0.5	0.5	0.5	**−**	**−**	**−**	**+**	*bla*_TEM_	1, 50	IncN
613-3	8	0.25	0.06	2	4	4	2	0.5	0.5	0.25	**−**	+(S1)	**−**	**−**	**−**	1, 60	UT
906-4	>256	0.25	0.06	4	2	8	2	0.12	0.25	0.25	**−**	**−**	**−**	**+**	*bla*_TEM_	1, 70	IncFII
1019-4	>256	16	1	4	2	>256	2	0.25	0.5	>256	+(B2)	**−**	**−**	**−**	*bla*_TEM_, *bla*_SHV-12_	1, >90	IncHI2
1377-3	256	1	0.12	8	128	>256	1	0.5	0.5	0.25	+(B4)	**−**	**+**	**−**	*bla*_TEM_, *bla*_DHA-1_	1, >90	UT
1426-4	64	4	0.25	32	2	4	2	0.25	0.5	0.25	+(B4)	**−**	**−**	**−**	*bla*_DHA-1_	1, 90	UT
1426-9	4	0.5	0.06	4	2	4	2	1	1	>256	**−**	+(S1)	**−**	**−**	**−**	1, 50	IncN
1516-3	4	0.25	0.06	2	2	8	2	0.5	1	>256	**−**	+(S1)	**−**	**−**	**−**	1, 50	IncN
1649-2	64	2	0.25	16	1	16	2	0.5	0.5	>256	+(B4)	**−**	**−**	**−**	**−**	1, 80	IncFII
1706-2	>256	0.25	0.06	2	32	4	1	0.5	1	0.25	**−**	+(S1)	**−**	**−**	*bla*_TEM_	1, 80	IncFII
1763-5	>256	0.25	0.03	4	1	>256	2	1	1	>256	**−**	+(S1)	**−**	**−**	*bla*_TEM_	1, 90	IncFII,
1962-1	64	2	0.25	32	1	4	2	0.25	0.5	0.25	+(B4)	**−**	**−**	**−**	*bla*_DHA-1_	1, 90	IncHI2
1962-2	4	0.25	0.06	4	64	32	2	0.25	0.25	>256	**−**	**−**	**+**	**−**	**−**	1, 50	IncN
1962-3	64	2	0.25	16	64	32	4	0.5	0.5	>256	+(B4)	**−**	**+**	**−**	*bla*_DHA-1_	2, 50/90	IncHI2, IncN

Abbreviations: AMP, ampicillin; CAZ, ceftazidime; CTX, cefotaxime; FOX, cefoxitin; TET, tetracycline; KN, kanamycin; FOS, fosfomycin; CIP, ciprofloxacin; LVX, levofloxacin; TMP, trimethoprim.

^a^Number and size of plasmids were predicted by the Kato & Liu method with modification^9^.
