# Supplementary material for: Plasmid-mediated quinolone resistance determinants in quinolone-resistant Escherichia coli isolated from patients with bacteremia in a university hospital in Taiwan, 2001–2015
Source: Sci Rep. 2016 Aug 30;6:32281. doi: 10.1038/srep32281 (PMC5004128; doi:10.1038/srep32281)
Supplement: Supplementary Information [file srep32281-s1.doc]

**Plasmid-mediated quinolone resistance determinants in quinolone-resistant *Escherichia coli* isolated from patients with bacteremia in a university hospital in Taiwan, 2001-2015**

Cheng-Yen Kao^1^, Hsiu-Mei Wu^1^, Wei-Hung Lin^2,3^, Chin-Chung Tseng^3^, Jing-Jou Yan^4^, Ming-Cheng Wang^3,5^, Ching-Hao Teng^6^, Jiunn-Jong Wu^1,7*^

^1^Department of Medical Laboratory Science and Biotechnology, College of Medicine, National Cheng Kung University, Tainan, Taiwan; ^2^Institute of Clinical Medicine, College of Medicine, National Cheng Kung University, Tainan, Taiwan; ^3^Division of Nephrology, Department of Internal Medicine, National Cheng Kung University Hospital, College of Medicine, National Cheng Kung University, Tainan, Taiwan; ^4^Department of Pathology, National Cheng Kung University Hospital, National Cheng Kung University, Tainan, Taiwan; ^5^Institute of Clinical Pharmacy and Pharmaceutical Sciences, College of Medicine, National Cheng Kung University, Tainan, Taiwan; ^6^Institute of Molecular Medicine, College of Medicine, National Cheng Kung University, Tainan, Taiwan; ^7^Department of Biotechnology and Laboratory Science in Medicine, School of Biomedical Science and Engineering, National Yang Ming University, Taipei, Taiwan.

**Supplementary Table S1. Oligonucleotide primers used in this study.**

| **Primer** | **Sequence (5’-3’)** | **Reference** |
| --- | --- | --- |
| **β-lactamase genes screening and sequencing** | |  |
| *bla*_CTX-M-13_ | GGTTAAAAAATCACTGCGTC | 1 |
|  | TTGGTGACGATTTTAGCCGC |  |
| *bla*_CTX-M-9_ | ATGGTGACAAAGAGAGTGCA | 1 |
|  | CCCTTCGGCGATGATTCTC |  |
| *bla*_TEM_ | CCCCTATTTGTTTATTTTTCT | 2 |
|  | GACAGTTACCAATGCTTAAT |  |
| *bla*_SHV_ | CCGGGTTATTCTTATTTGTC | 3 |
|  | TAGCGTTGCCAGTGCTCGAT |  |
| *bla*_DHA_ | ACACTGATTTCCGCTCTGCT | This study |
|  | ACAATCGCCACCTGTTTTTC |  |
| *bla*_CMY_ | CTGACAGCCTCTTTCTCCACA | This study |
|  | CTACGTAGCTGCCAAATCCAC |  |
| **PMQR genes screening and sequencing** | | |
| aac(6′)Ib-cr | TTG CGA TGC TCT ATG AGT GGC TA | 4 |
|  | CTC GAA TGC CTG GCG TGT TT |  |
| *oqxA* | GAC AGC GTC GCA CAG AAT G | 5 |
|  | GGA GAC GAG GTT GGT ATG GA |  |
| *oqxB* | CGA AGA AAG ACC TCC CTA CCC | 5 |
|  | CGC CGC CAA TGA GAT ACA |  |
| *qepA* | GCA GGT CCA GCA GCG GGT AG | 6 |
|  | CTT CCT GCC CGA GTA TCG TG |  |
| *qnrA* | TTCAGCAAGAGGATTTCTCA | 7 |
|  | GGCAGCACTATTACTCCCAA |  |
| *qnrB* | CCTGAGCGGCACTGAATTTAT | 7 |
|  | GTTTGCTGCTCGCCAGTCGA |  |
|  | GTTGGCGAAAAAATTGACAGAA |  |
|  | TTTGCAAGGCGTCAAACTGG |  |
|  | ACTCCGAATTGGTCAGATCG |  |
| *qnrC* | GGGTTGTACATTTATTGAATC | 8 |
|  | TCCACTTTACGAGGTTCT |  |
| *qnrD* | CGG GGA ATA GAG TTA AAA AT | 9 |
|  | TAT CGG TGA ACA ATA ACA CC |  |
| *qnrS* | CAATCATACATATCGGCACC | 10 |
|  | TCAGGATAAACAACAATACCC |  |
| *qnrVC* | AATTTTAAGCGCTCAAACCTCCG | 11 |
|  | TCCTGTTGCCACGAGCATATTTT |  |
| **QRDR amplification and sequencing** | | |
| *gyrA* | GCGATGTCGGTCATTGTT | 12 |
|  | ACTTCCGTCAGGTTGTGC |  |
| *parC* | TGCGTTGCCGTTTATTGG | 12 |
|  | GCAGGTTATGCGGTGGAAT |  |
| **Phylogenetic grouping** | | |
| ChuA.1 | GACGAACCAACGGTCAGGAT | 13 |
| ChuA.2 | TGCCGCCAGTACCAAAGACA |  |
| YjaA.1 | TGAAGTGTCAGGAGACGCTG | 13 |
| YjaA.2 | ATGGAGAATGCGTTCCTCAAC |  |
| TspE4C2.1 | GAGTAATGTCGGGGCATTCA | 13 |
| TspE4C2.2 | CGCGCCAACAAAGTATTACG |  |
| AceK.f | AACGCTATTCGCCAGCTTGC | 14 |
| ArpA1.r | TCTCCCCATACCGTACGCTA |  |
| ArpAgpE.f | GATTCCATCTTGTCAAAATATGCC | 14 |
| ArpAgpE.r | GAAAAGAAAAAGAATTCCCAA GAG |  |
| **Plasmid replicon typing** | | |
| repI | CGAAAGCCGGACGGCAGAA | 15 |
|  | TCGTCGTTCCGCCAAGTTCGT |  |
| ardA | ATGTCTGTTGTTGCACCTGC | 15 |
|  | TCACCGACGGAACACATGACC |  |
| trbA | CGACAAATGCTTCCGGGGT | 15 |
|  | TCTTACAATCGACAGCCTGT |  |
|  | CGAATCCCTCACCATCCAG |  |
| sogS | TTCCGGGGCGTAGACAATACT | 15 |
|  | AACAGTGATATGCCGTCGC |  |
| pilL | CCATATGACCATCCAGTGCG | 15 |
|  | AACCACTATCTCGCCAGCAG |  |
| smr0018 | ATAATGATTCACCGGGGTAG | 16 |
|  | CTTCAGGCTATCGTTTCG |  |
| smr0199 | TGTTTACACCACCAGCAG | 16 |
|  | TTTAACAACAGGAGTCGGG |  |
| FII | CTGATCGTTTAAGGAATTTT | 17 |
|  | CACACCATCCTGCACTTA |  |
| FIA | CCATGCTGGTTCTAGAGAAGGTG | 17 |
|  | GTATATCCTTACTGGCTTCCGCAG |  |
| FIB | TCTGTTTATTCTTTTACTGTCCAC | 17 |
|  | CTCCCGTCGCTTCAGGGCATT |  |
| FIC | GTGAACTGGCAGATGAGGAAGG | 17 |
|  | TTCTCCTCGTCGCCAAACTAGAT |  |
| repN | GTCTAACGAGCTTACCGAAG | 18 |
|  | ACGGTCATTTAACCAAGCATG |  |
| traJ | CTTCTTCCATAGTTACTGTGCT | 18 |
|  | CATCCACGGCTAAATACCTG |  |
| korA | GGAACGTTTGTAYCTTGTATTG | 18 |
|  | ACTCACTATCTTCTGTTGATTG |  |
|  |  |  |

**References**

1. Saladin, M. *et al*. Diversity of CTX-M beta-lactamases and their promoter regions from *Enterobacteriaceae* isolated in three Parisian hospitals. *FEMS Microbiol Lett*. **209**, 161-168 (2002).
2. Yan, J. J., Ko, W. C. & Wu, J. J. Identification of a plasmid encoding SHV-12, TEM-1, and a variant of IMP-2 metallo-beta-lactamase, IMP-8, from a clinical isolate of *Klebsiella pneumoniae*. *Antimicrob Agents Chemother*. **45**, 2368-2371 (2001).
3. Nuesch-Inderbinen, M. T., Hachler, H. & Kayser, F. H. Detection of genes coding for extended-spectrum SHV beta-lactamases in clinical isolates by a molecular genetic method, and comparison with the E test. *Eur J Clin Microbiol Infect Dis*. **15**, 398-402 (1996).
4. Park, C. H., Robicsek, A., Jacoby, G. A., Sahm, D. & Hooper, D. C. Prevalence in the United States of *aac(6')-Ib-cr* encoding a ciprofloxacin-modifying enzyme. *Antimicrob Agents Chemother*. **50**, 3953-3955 (2006).
5. Chen, X. *et al*. Prevalence of *qnr*, *aac(6')-Ib-cr*, *qepA*, and *oqxAB* in *Escherichia coli* isolates from humans, animals, and the environment. *Antimicrob Agents Chemother*. **56**, 3423-3427 (2012).
6. Cattoir, V., Poirel, L. & Nordmann, P. Plasmid-mediated quinolone resistance pump QepA2 in an *Escherichia coli* isolate from France. *Antimicrob Agents Chemother*. **52**, 3801-3804 (2008).
7. Wu, J. J., Ko, W. C., Wu, H. M. & Yan, J. J. Prevalence of Qnr determinants among bloodstream isolates of *Escherichia coli* and *Klebsiella pneumoniae* in a Taiwanese hospital, 1999-2005. *J Antimicrob Chemother*. **61**, 1234-1239 (2008).
8. Wang, M. *et al*. New plasmid-mediated quinolone resistance gene, *qnrC*, found in a clinical isolate of *Proteus mirabilis*. *Antimicrob Agents Chemother*. **53**, 1892-1897 (2009).
9. Cavaco, L. M., Hasman, H., Xia, S. & Aarestrup, F. M. *qnrD*, a novel gene conferring transferable quinolone resistance in *Salmonella* enterica serovar *Kentucky* and *Bovismorbificans* strains of human origin. *Antimicrob Agents Chemother*. **53**, 603-608 (2009).
10. Wu, J. J., Ko, W. C, Tsai, S. H. & Yan, J. J. Prevalence of plasmid-mediated quinolone resistance determinants QnrA, QnrB, and QnrS among clinical isolates of *Enterobacter cloacae* in a Taiwanese hospital. *Antimicrob Agents Chemother*. **51**, 1223-1227 (2007).
11. Kim, H. B. *et al*. Transferable quinolone resistance in *Vibrio cholerae*. *Antimicrob Agents Chemother*. **54**, 799-803 (2010).
12. Zhao, L. *et al*. Molecular epidemiology and genetic diversity of fluoroquinolone-resistant *Escherichia coli* isolates from patients with community-onset infections in 30 Chinese county hospitals. *J Clin Microbiol.* **53**, 766-770 (2015).
13. Clermont, O., Bonacorsi, S. & Bingen, E. Rapid and simple determination of the *Escherichia coli* phylogenetic group. *Appl Environ Microbiol*. **66**, 4555-4558 (2000).
14. Clermont, O., Christenson, J. K., Denamur, E. & Gordon, D. M. The Clermont *Escherichia coli* phylo-typing method revisited: improvement of specificity and detection of new phylo-groups. *Environ Microbiol Rep*. **5**, 58-65 (2013).
15. Garcia-Fernandez, A. *et al*. Multilocus sequence typing of IncN plasmids. *J Antimicrob Chemother*. **66**, 1987-1991 (2008).
16. Garcia-Fernandez, A. *et al*. Multilocus sequence typing of IncI1 plasmids carrying extended-spectrum beta-lactamases in *Escherichia coli* and *Salmonella* of human and animal origin. *J Antimicrob Chemother*. **61**, 1229-1233 (2008).
17. Villa, L., Garcia-Fernandez, A., Fortini, D. & Carattoli, A. Replicon sequence typing of IncF plasmids carrying virulence and resistance determinants. *J Antimicrob Chemother.* **65**, 2518-2529 (2010).
18. Garcia-Fernandez, A. & Carattoli, A. Plasmid double locus sequence typing for IncHI2 plasmids, a subtyping scheme for the characterization of IncHI2 plasmids carrying extended-spectrum beta-lactamase and quinolone resistance genes. *J Antimicrob Chemother*. **65**, 1155-1161 (2010).
